# High Wear Resistance of POSS Grafted-Polyimide/Silica Composites under Atomic Oxygen Conditions

**DOI:** 10.3390/polym15102385

**Published:** 2023-05-19

**Authors:** Huimin Qi, Yang Lei, Xuemei Lei, Ding Zhang, Yafeng Zhang, Jiaxin Yu, Baogang Guo

**Affiliations:** 1 Key Laboratory of Testing Technology for Manufacturing Process, Ministry of Education, Southwest University of Science and Technology, Mianyang 621010, China; leiyang@mails.swust.edu.cn (Y.L.); studentzyf1@163.com (Y.Z.); yujiaxin@swust.edu.cn (J.Y.); 2 School of Materials Science and Engineering, National Institute for Advanced Materials, Nankai University, Tongyan Road 38, Tianjin 300350, China; zhangding@nankai.edu.cn

**Keywords:** polyimide, NH_2_-POSS, tribological properties, atomic oxygen, tribofilm

## Abstract

Polyimide-bearing retainer has been successfully used in space environment. However, the structural damage of polyimide induced by space irradiation limits its wide use. In order to further improve the atomic oxygen resistance of polyimide and comprehensively investigate the tribological mechanism of polyimide composites exposed in simulate space environment, 3-amino-polyhedral oligomeric silsesquioxane (NH_2_-POSS) was incorporated into a polyimide molecular chain and silica (SiO_2_) nanoparticles were in situ added into polyimide matrix and the combined effect of vacuum environment, and atomic oxygen (AO) on the tribological performance of polyimide was studied using bearing steel as the counterpart by a ball on disk tribometer. XPS analysis demonstrated the formation of protective layer induced by AO. The wear resistance of polyimide after modification was enhanced under AO attack. FIB-TEM confirmed that the inert protective layer of Si was formed on the counterpart during the sliding process. Mechanisms behind this are discussed based on the systematic characterization of worn surfaces of the samples and the tribofilms formed on the counterbody.

## 1. Introduction

Lubrication failure of moving parts in spacecraft induced by vacuum, atomic oxygen irradiation and high/low temperature, etc., is an issue needing urgent attention in space environment [1,2,3,4,5,6]. When most materials are exposed to vacuum environment, mass loss and performance degradation occur [7,8]. For example, the direct contact sliding metal surfaces are prone to adhesion and even cold welding; therefore, the sliding surfaces was needed to lubricate. Generally, the volatilization and sublimation can happen to the conventional liquid lubricant, leading to the weakened lubrication [9]. Moreover, the volatile components may pollute the precision instruments in the spacecraft. Some lubricating oils and greases will creep, that is, the lubricant will migrate out of the surface, resulting in insufficient lubricant on the working face [9,10]. In this case, solid lubricating materials such as polytetrafluoroethylene, polyimide, molybdenum disulfide and silicon dioxide have received wide-ranging attention, which exhibit excellent tribological performance in vacuum environment [6,11,12,13,14]. On the other hand, the main component of the space environment in low earth orbit is atomic oxygen (AO), the content of which can reach 80%. As is well-known, the atomic oxygen exhibits strong oxidizability, which can significantly affect the performance of solid lubricant and further reduce the reliability and service life of spacecraft [15,16,17].

Polymer self-lubricating materials are used as crucial moving components of spacecraft in space systems, due to their good mechanical performance, high wear resistance, resistance to irradiation and chemical inertness [15,16,18,19,20,21]. Polyimide (PI) as one of the high performance engineering thermoplastic polymers has been used in space environment. Recently, most researchers are focusing on the modification of structure and composition of polyimide in order to improve its performance used in space environment [1,22,23,24,25]. Song et al. [22] coated the polydimethylsiloxane/polyhedral oligomeric silsesquioxanes hybrid materials on the polyimide films. The result indicated that a protected barrier growth from the polydimethylsiloxane/polyhedral oligomeric silsesquioxanes coating prevented the degradation of the PI film exposed to atomic oxygen environment. Vernigorov et al. [23] found that polyimide modified by hyperbranched polyorganosiloxanes showed excellent resistance to the action of atomic oxygen. Minton et al. [1] reported that the incorporation of polyhedral oligomeric silsesquioxane (POSS) into the polyimide matrix could improve the resistant to atomic-oxygen attack by formation of a silica passivation layer. In the basis of above results, it can be concluded that the modification of PI by silicone material show excellent resistance to AO irradiation.

Furthermore, it was found that the nanoparticles used as the fillers of polymer-matrix could not only improve the resistance to AO irradiation of composites, but also enhance their mechanical and tribological performance [15,16,22,24]. As reported by researchers, the nanoparticles such as silicon dioxide (SiO_2_), titanium dioxide (TiO_2_) and zirconium dioxide (ZrO_2_) could enhance the AO resistance of composites by formation of a protective layer [25,26,27]. Owing to the cost-effectiveness and high hardness and compressive strength, SiO_2_ is a commonly used anti-wear additive. Bharadwaja et al. [28] reported that the properties of the epoxy matrix with the introduction of SiO_2_, hardness and impact strength, also the wear and the friction coefficient of the nanocomposite were significantly reduced by adding 1% of the volume of SiO_2_ nanoparticles to the epoxy matrix. In general, the friction and wear of polymeric composites were also improved by the formation of nano-based tribofilm during the sliding process. Lv et al. [29] found that the nano-ZrO_2_ reinforced polyimide composites obviously decreased the mass loss induced by atomic oxygen irradiation, compared with pure polyimide. Liu et al. [26] reported that the incorporation of SiO_2_ could improve the AO resistance of PI film. However, the tribological behaviors of PI composites under in situ vacuum atomic oxygen irradiation and vacuum environment have rarely been reported and the associated mechanism underlying the tribological behaviors is also not well-understood. Therefore, there is a constant requirement to develop excellent self-lubricated PI composites with superior tolerance to atomic oxygen irradiation conditions.

Herein, silica nanoparticles were in situ added into 3-amino-polyhedral oligomeric silsesquioxane modified polyimide molecular and the effects of atomic oxygen irradiation on the structural, mechanical, and tribological properties of these composites were studied. Moreover, the friction and wear of PI and its composites were compared under air, vacuum and vacuum atomic oxygen irradiation conditions. The morphologies of the worn surfaces of PI composites and the tribofilms, and the related tribo-chemical reactions induced by atomic oxygen irradiation were comprehensively characterized. It is hoped that this work provides huge amounts of accurate data for aircraft design subjected to atomic oxygen irradiation conditions.

## 2. Materials and Methods

### 2.1. Materials

Pyromellitic dianhydride (PMDA), 4,4′-diamino-diphenyl ether (ODA), N-methylpyrrolidone (NMP) and 3-aminopropyltriethoxysilane were purchased from Shanghai Aladdin Bio-Chem Technology Co., Ltd., Shanghai, China. Tetrahydrofuran (THF) and hydrochloric acid (HCl) were purchased from Chengdu Cologne Chemicals Co., Ltd., Chengdu, China. Nano-silica (20 nm) was provided by Nanjing Tianxing New Materials Co., Ltd., Nanjing, China.

### 2.2. Synthesis of Polyimide Composites

**Figure 1:**
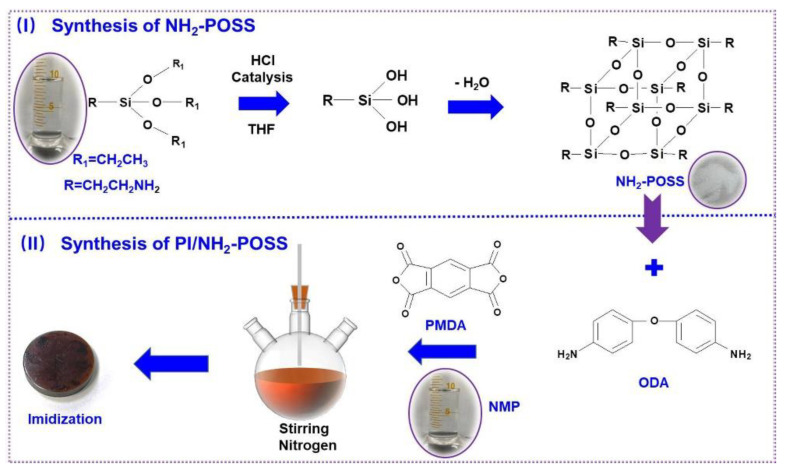
Schematic illustration of PI/NH_2_-POSS.

Synthesis of 3-amino-polyhedral oligomeric silsesquioxane (NH_2_-POSS): the NH_2_-POSS was prepared via dehydration condensation method [30], as shown in Figure 1. Briefly, 20 mL THF and 20 mL 3-aminopropyltriethoxysilane were added into a 100 mL three-necked flask, and 5 mL HCl used as reaction catalyst was then added. After reacting at room temperature for 72 h, the white crystals were obtained. Finally, the product was washed with alcohol, and oven-dried at 60 °C.

Four types of PI coatings were used, i.e., pure PI, PI modified with NH_2_-POSS (PI/NH_2_-POSS), PI filled with SiO_2_ (PI/SiO_2_), and PI/NH_2_-POSS filled with SiO_2_. The detailed compositions of PI samples are shown in Table 1. Figure 1 presents the schematic illustration of PI/NH_2_-POSS. First, 0.9 g ODA, 10 mL NMP solvent and 0.018 g NH_2_-POSS were added to a 100 mL three-neck flask. After stirring 2 h, the monomer was dissolved. Then, 0.9 g PMDA was gradually added to the above solution in ice water bath and nitrogen atmosphere, and stirred for 24 h. Viscous PAA/NH_2_-POSS solution was obtained and then coated on the bearing steel surface (GCr15). The sample was subsequently placed on a heating platform, 80 °C drying for 4 h and then placed in a temperature-programmable oven; the temperature was set as 100 °C, 200 °C, 260 °C for 60 min, respectively. Finally, the PI/NH_2_-POSS sample was obtained. The preparation method of PI/SiO_2_ and PI/NH_2_-POSS/SiO_2_ was almost similar with that of PI/NH_2_-POSS. The only modification was replacing NH_2_-POSS with other fillers.

### 2.3. Atomic Oxygen Irradiation and Tribology Tests

Tribological test in air, high-vacuum environments and vacuum atomic oxygen irradiation were carried out in a ground-based simulation system established at the Lanzhou Institute of Physics (Lanzhou, China) and the schematic of vacuum tribometer is provided in Figure S1, which is referred from the research group in which I pursued my doctoral degree. The high-vacuum environment was 1.0 × 10^−3^ Pa. The flux of AO beam was 5.3 × 10^15^ atoms/cm^2^·s^−1^ determined via the standard method of Kapton mass loss and the vacuum of the environment was 1.8 × 10^−1^ Pa. In this study, every sample was sliding under in situ irradiation condition. The tribological performances of the samples were investigated using a ball-on-disk test. The ball with ø = 6.0 mm was made of GCr15 steel and the disk with ø = 24.0 mm was covered with the materials. The applied load was set as 5 N using the weights and the sliding speed was 0.126 m/s according to the reference [29]; the duration of each test lasted 30 min and only one specimen was conducted for each condition. Prior to tribology test, GCr15 counterpart was washed with acetone for 30 min. The friction coefficient was recorded online, and the errors were calculated with the average of any three points at the end of the sliding process. The width of the wear mark was measured using an optical microscope (U-25ND25, OLYMPUS, T2, Tokyo, Japan), and the wear rate was calculated according to the following formula:

(1) $$W_{s}=\left[\frac{\pi r^{2}}{180}arcsin\left(\frac{b}{2r}\right)-\frac{b}{2}\sqrt{r^{2}-\frac{b^{2}}{4}}\right]\pi d/PL$$

where *Ws* is the wear rate (mm^3^/Nm) and *r* is the radius of the dual steel ball (m), *b* is the width of the wear mark (m), *d* is the rotating diameter, *P* represents the applied load (N), *L* corresponds to the sliding distance (m) in total. The error of the wear rate was calculated via three different wear marks of the worn surface of each sample. Additionally, the roughness (Ra) of the GCr15 balls were measured via white light scanning profilometry (MFT-3000, Rtec, San Jose, CA, USA) before and after sliding.

### 2.4. Characterization of Polyimide Composites

The nano-mechanical properties of the samples before and after atomic oxygen irradiation were characterized using a Nano Indenter (G200, Keysight, Santa Rosa, CA, USA), and the maximum indentation depth was set to 350 nm. Additionally, the chemical structures in the pure polyimide and composite samples before and after atomic oxygen irradiation were characterized via Fourier transform-infrared spectroscopy (FTIR, Nicolet-5700, Thermo Fisher, Waltham, MA, USA) and X-ray photoelectron spectroscopy (XPS, 250Xi, ESCALAB, Waltham, MA, USA). Thermogravimetric analyses (TGA, TGA 8000, PerkinElmer, Waltham, MA, USA) of all the samples before and after AO attack were conducted under nitrogen flow with a heating rate of 10 °C/min. Moreover, images of all the samples were captured with a scanning electron microscope (SEM, Ultra 55, Zeiss, Oberkochen, Germany).

### 2.5. Characterization of Worn Surfaces and Tribofilms

The worn surfaces of the composites were characterized using an optical microscope (U-25ND25, OLYMPUS, T2,Tokyo, Japan). Nanostructures of the tribofilms were imaged with a scanning electron microscope (SEM, 7610F, JEOL, Tokyo, Japan). Additionally, the changes in the chemical states of the tribofilms were analyzed with X-ray photoelectron spectroscopy (XPS, 250Xi, ESCALAB, Waltham, MA, USA). Moreover, slices of the tribofilm were prepared using focused ion beam (FIB) machining (Quanta 3D FEG, FEI, Hillsboro, OR, USA), and the fractured surfaces were then observed via high-resolution transmission electron microscopy (HR-TEM, TecnaiG2 TF20, FEI, Hillsboro, OR, USA).

## 3. Results and Discussions

### 3.1. Mechanical and Structure Analysis

**Figure 2:**
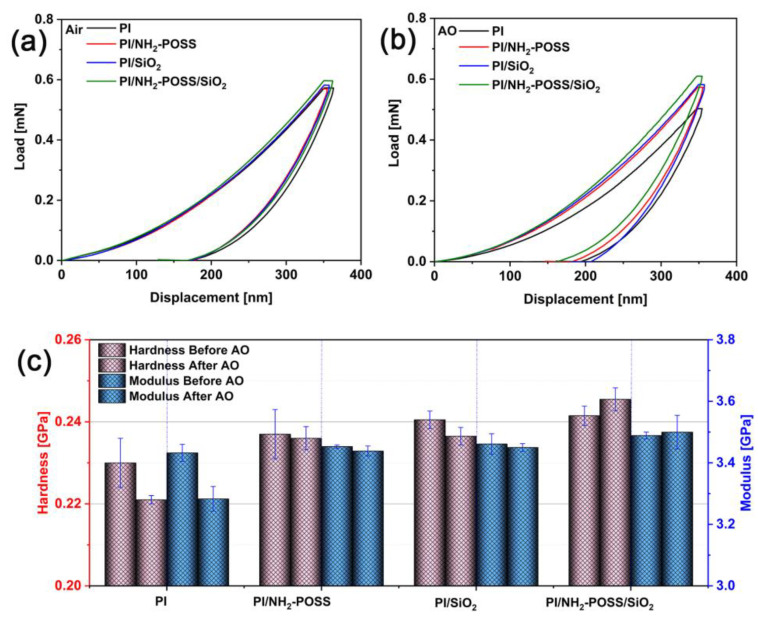
The load-depth curves (**a**) before AO attack, (**b**) after AO attack, (**c**) modulus and hardness of PI materials before and after AO attack, respectively.

In order to explore the influence of atomic oxygen irradiation on the mechanical properties of polyimide composites, nano-indentation tests were performed to determine the hardness and modulus of the samples. As shown in Figure 2a,b, the load-depth curves measured during the nano-indentation tests were different before and after AO irradiation. It could be seen that the applied loads were approximately 0.58 and 0.50 mN for PI before and after AO irradiation, respectively, when they had the same indentation depth. The corresponding hardness and modulus of pure PI were 0.230 GPa and 3.42 GPa, respectively, which were reduced to 0.221 GPa and 3.28 GPa after AO irradiation (Figure 2c). We believed that the PI was degraded, induced via a serious AO attack. Regarding PI/NH_2_-POSS and PI/SiO_2_, their elastic recoveries became low after AO attack. Meanwhile, the hardness and the modulus of PI/NH_2_-POSS and PI/SiO_2_ were also reduced. However, their resistance to AO was enhanced via incorporation of SiO_2_ or NH_2_-POSS comparing with pure PI. For PI/NH_2_-POSS/SiO_2_, the applied load and the elastic recovery increased after AO irradiation, and the hardness and modulus were enhanced from 0.241 GPa to 0.250 GPa and 3.49 GPa to 3.50 GPa, respectively (Figure 2c). Therefore, it was believed that a protective layer growing from SiO_2_ and NH_2_-POSS prevents these from AO attack.

**Figure 3:**
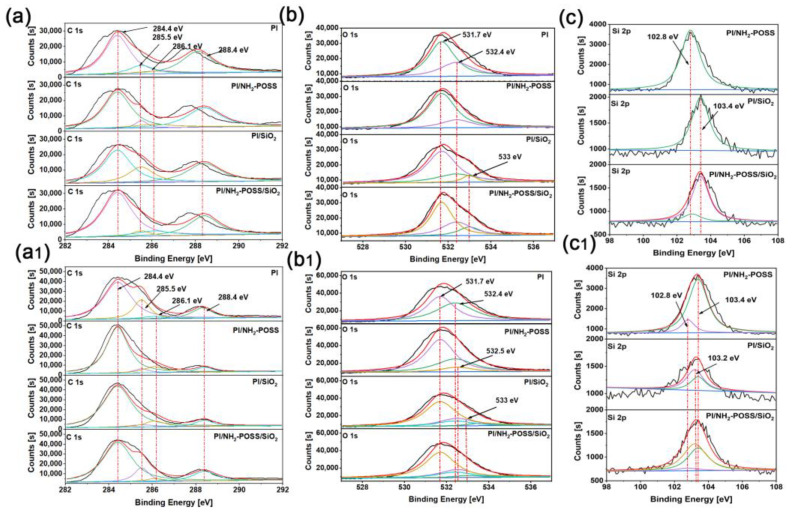
XPS spectra of (**a**,**a1**) C 1s, (**b**,**b1**) O 1s, (**c**,**c1**) Si 2p of PI, PI/NH_2_-POSS, PI/SiO_2_ and PI/NH_2_-POSS/SiO_2_ before and after atomic oxygen irradiation, respectively.

To confirm the AO irradiation-induced changes in polyimide before and after modification, XPS data were determined for all the samples. The chemical state of C, O and Si in PI, PI/NH_2_-POSS, PI/SiO_2_ and PI/NH_2_-POSS/SiO_2_ is provided in Figure 3. The peaks of C-C, C-N, C-O, and C=O groups in the PI corresponded to 284.4 eV, 285.5 eV, 286.1 eV, and 288.4 eV in the C1s spectra, respectively [31]. The C-O and C=O groups were further determined at 532.4 eV and 531.7 eV in O1s spectra, respectively. It was found that the intensity of C 1s and O 1s was obviously changed after AO attack. Obviously, the intensity of C=O and C-O was enhanced after AO irradiation as shown in Figure 3(a,a1), while that of C-C was decreased. Zhao et al. [32] reported that partial carbon in PI was released in the forms of CO_2_ and CO when polyimide was exposed to AO environment; on the other hand, the polyimide was partly oxidized to produce C=O, increasing the O content. Judging from O 1s (Figure 3(b,b1)), the intensity of O 1s in PI/NH_2_-POSS, PI/SiO_2_ and PI/NH_2_-POSS/SiO_2_ after atomic oxygen irradiation was not increased significantly compared with PI and PI/NH_2_-POSS in air, indicating the excellent resistance to atomic oxygen irradiation of PI composites filled with SiO_2_.

With regard to Si 2p (Figure 3(c,c1)), the intensity of Si in PI/NH_2_-POSS before and after AO attack showed insignificant difference, while that in PI/SiO_2_ and PI/NH_2_-POSS/SiO_2_ decreased after AO attack. Before AO attack, the peak of Si-O in NH_2_-POSS was confirmed at 102.8 eV [22], and the peak of Si-O in SiO_2_ was confirmed at 103.4 eV [33]. Moreover, the peaks at 102.8 eV and 103.4 eV appeared simultaneously in PI/NH_2_-POSS/SiO_2_. After AO attack, the Si-O could also be confirmed in PI/NH_2_-POSS, PI/SiO_2_ and PI/NH_2_-POSS/SiO_2_. Nevertheless, the new peak at 103.2 eV appeared in PI/SiO_2_ and PI/NH_2_-POSS/SiO_2_, which corresponded to the Si(O)x formed after AO attack [22].

**Figure 4:**
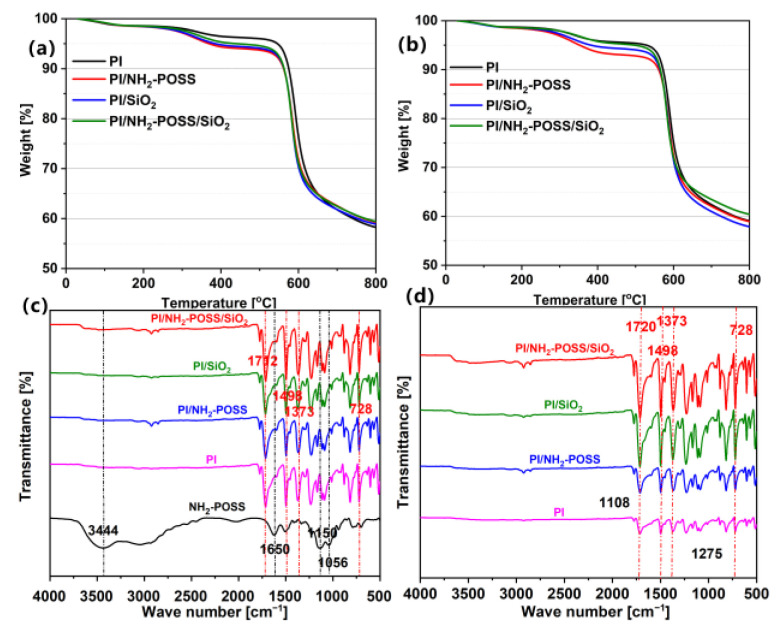
Thermogravimetric curves of PI, PI/NH_2_-POSS, PI/SiO_2_ and PI/NH_2_-POSS/SiO_2_ (**a**) before and (**b**) after AO attack; FTIR-ATR spectra of NH_2_-POSS, PI, PI/NH_2_-POSS, PI/SiO_2_ and PI/NH_2_-POSS/SiO_2_ (**c**) before and (**d**) after AO attack.

Figure 4a,b shows the thermal stability before and after AO attack, respectively. It can be seen that the starting decomposition (~5 wt % weight loss) of PI, PI/NH_2_-POSS, PI/SiO_2_ and PI/NH_2_-POSS/SiO_2_ was at 550 °C, 373 °C, 385 °C and 461 °C before AO attack, respectively. However, that of PI, PI/NH_2_-POSS, PI/SiO_2_ and PI/NH_2_-POSS/SiO_2_ was reduced to 489 °C, 350 °C, 380 °C and 453 °C, respectively, after AO attack, indicating the slight destruction of the samples induced by AO attack. The fastest weight loss happened at almost the same temperature of ~600 °C for all samples. Thus, all the samples exhibited great thermal stability although they were attacked by atomic oxygen.

Figure 4c gives the FTIR spectrum of NH_2_-POSS, PI, PI/NH_2_-POSS, PI/SiO_2_ and PI/NH_2_-POSS/SiO_2_. The absorption bands of N-H at 3444 cm^−1^ and 1650 cm^−1^ were confirmed, and the peaks at 1150 cm^−1^ and 1056 cm^−1^ were attributed to Si-C stretching and the symmetric stretching of Si-O-Si bond in NH_2_-POSS, respectively. The result indicated that NH_2_-POSS was successfully prepared. Additionally, the chemical structure changes of PI, PI/NH_2_-POSS, PI/SiO_2_ and PI/NH_2_-POSS/SiO_2_ were investigated via FTIR. As shown in Figure 4c, the characteristic bands at 1712 cm^−1^, 1373 cm^−1^ and 728 cm^−1^ corresponded to C=O asymmetry stretching vibrations, C-N-C stretching vibrations and C=O bending vibrations in PI, respectively [34]. The characteristic absorption peak of benzene ring was confirmed at 1498 cm^−1^. With regard to PI/NH_2_-POSS, PI/SiO_2_ and PI/NH_2_-POSS/SiO_2_, the characteristic absorption peaks were almost consistent with that of PI. After AO irradiation, the absorption peak intensity was significantly reduced for PI and PI/NH_2_-POSS as shown in Figure 4d. Nevertheless, the intensity of PI/SiO_2_ and PI/NH_2_-POSS/SiO_2_ showed little difference compared with that before AO irradiation, due to the excellent atomic oxygen resistance of SiO_2_.

**Figure 5:**
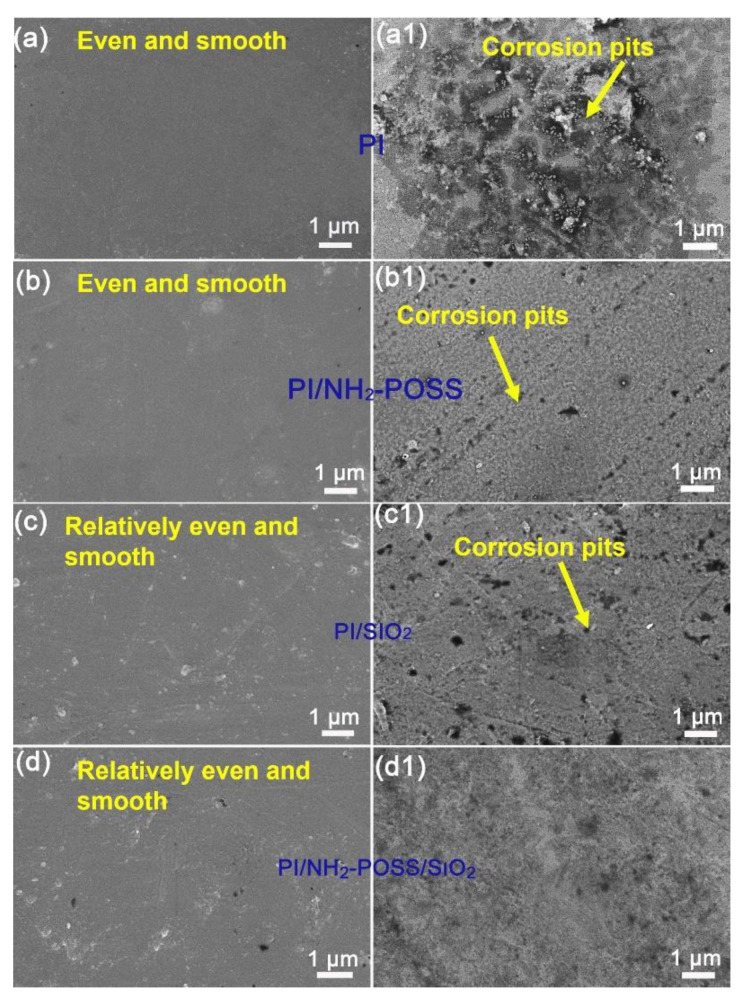
SEM micrographs of (**a**,**a1**) PI, (**b**,**b1**) PI/NH_2_-POSS, (**c**,**c1**) PI/SiO_2_ and (**d**,**d1**) PI/NH_2_-POSS/SiO_2_ PI before and after AO attack.

SEM was used to explore the effect of atomic oxygen attack on the surface morphologies of PI, PI/NH_2_-POSS, PI/SiO_2_ and PI/NH_2_-POSS/SiO_2_. It can be clearly found that the surfaces of all the samples were relatively even and smooth before AO attack (Figure 5a–d). The morphologies of PI, PI/NH_2_-POSS, PI/SiO_2_ and PI/NH_2_-POSS/SiO_2_ become rough and displayed flannelette blanket shape after AO attack (Figure 5(a1–d1)). In particular, the pure PI was seriously eroded as shown in Figure 5(a1), and many corrosion pits appeared in the PI surface as indicated by the yellow arrow. The modification of polyimide by NH_2_-POSS and SiO_2_ could induce the formation of an inert protective layer. Thus, the surfaces of PI/NH_2_-POSS and PI/SiO_2_ were relative even, and the depth of the corrosion pits became small (Figure 5(b1,c1)). Moreover, PI/NH_2_-POSS/SiO_2_ showed the best atomic oxygen resistance (Figure 5(d1)). It was believed that the synergistic effect of NH_2_-POSS and SiO_2_ could significantly inhibit the erosion from atomic oxygen.

### 3.2. Tribological Behaviors

**Figure 6:**
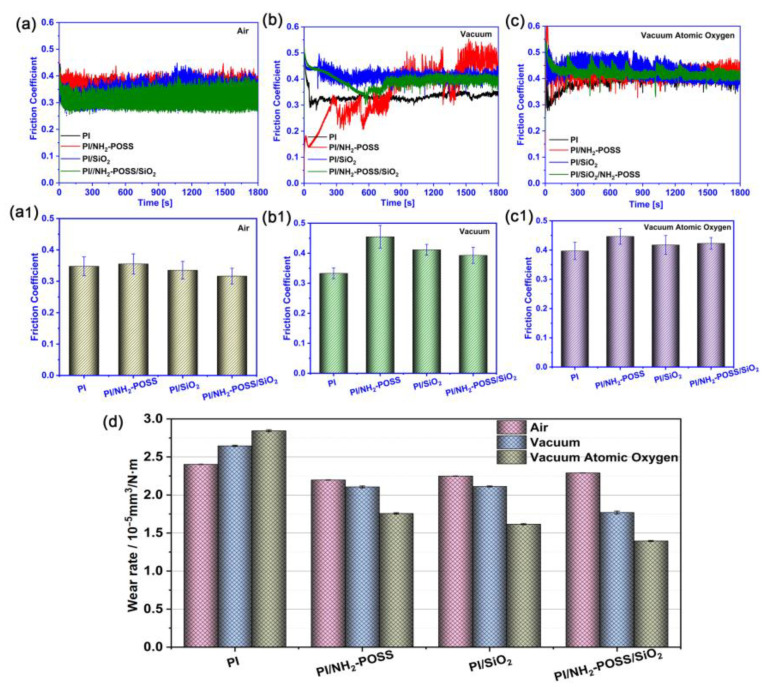
The friction coefficients evolution and its average values of PI, PI/NH_2_-POSS, PI/SiO_2_ and PI/NH_2_-POSS/SiO_2_ (**a**,**a1**) in air, (**b**,**b1**) vacuum environment, and (**c**,**c1**) vacuum atomic oxygen environment, respectively; and the (**d**) corresponding average wear rate.

Figure 6 shows the tribological properties of PI, PI/NH_2_-POSS, PI/SiO_2_ and PI/NH_2_-POSS/SiO_2_ when they were slid against steel counterparts under different conditions. In air, the friction coefficients were relatively unstable, and the modification showed little effect on their performance. The friction coefficients of all the samples were in the range of 0.2~0.40 in air (Figure 6(a,a1)). In a vacuum environment, the friction coefficients of PI/NH_2_-POSS and PI/NH_2_-POSS/SiO_2_ displayed great fluctuation (Figure 6b). In particular, the friction coefficient of PI/NH_2_-POSS kept increasing during all the sliding process and the running-in process of PI/NH_2_-POSS/SiO_2_ lasted approximately 600 s. However, the sliding process of pure PI was relatively stable. As shown in Figure 6b, the average friction coefficient of PI was stable at approximately 0.32, while the values of PI/NH_2_-POSS, PI/SiO_2_ and PI/NH_2_-POSS/SiO_2_ were 0.45, 0.41 and 0.39, respectively. It was believed that the modification of PI enhanced the interfacial interaction between the sliding surfaces, releasing a lot of friction heat. In a vacuum environment, the friction heat was difficult to transmit. In this case, adhesion easily occurred between the friction interfaces. When sliding occurred in the vacuum atomic oxygen environment, the friction coefficients of all the samples were higher than those obtained in the air and more stable than those in vacuum environment (Figure 6c). The average friction coefficients of PI, PI/NH_2_-POSS, PI/SiO_2_ and PI/NH_2_-POSS/SiO_2_ were 0.39, 0.44, 0.41 and 0.42, respectively. We believe that the polyimide structure was destroyed under AO environment, which contributed to the high friction coefficient [15,35]. Moreover, the friction heat in vacuum atomic oxygen environment was difficult to dissipate, which aggravated the harsh working conditions.

Regarding the wear rate, the modification of PI played an important role on their wear resistance under different conditions. It was found that pure PI displayed the terrible wear rate (Figure 6d). The highest wear rate of PI was 2.84 × 10^−5^ mm^3^/N·m in vacuum atomic oxygen environment. The modification of PI could improve its wear resistance. Moreover, the wear rates of PI/NH_2_-POSS, PI/SiO_2_ and PI/NH_2_-POSS/SiO_2_ in air were higher than those of vacuum and vacuum atomic oxygen environments. In vacuum atomic oxygen environment, PI/NH_2_-POSS, PI/SiO_2_ and PI/NH_2_-POSS/SiO_2_ displayed excellent wear resistance. Particularly, PI/NH_2_-POSS/SiO_2_ showed the lowest wear rate 1.32 × 10^−5^ mm^3^/N·m. We believed that the protective layer formed on the surface PI/NH_2_-POSS/SiO_2_ enhanced the load carry capability of the sliding interface.

**Figure 7:**
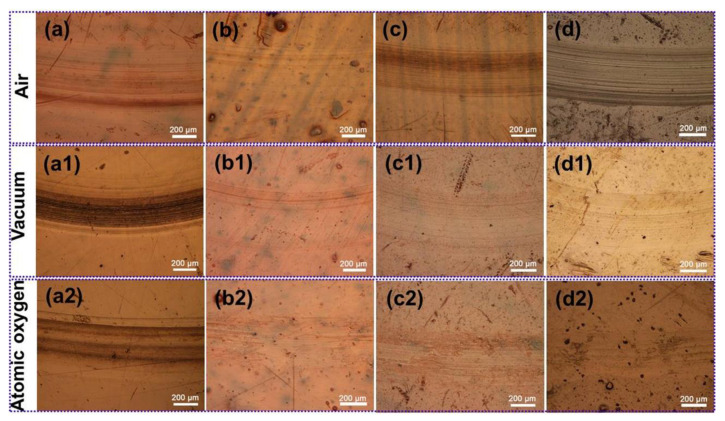
Optical images of the worn surfaces of (**a**–**a1**) PI, (**b**–**b2**) PI/NH_2_-POSS, (**c**–**c2**) PI/SiO_2_ and (**d**–**d2**) PI/NH_2_-POSS/SiO_2_ in air, vacuum environment, and vacuum atomic oxygen environment, respectively.

Figure 7 shows the optical images of worn surfaces of PI, PI/NH_2_-POSS, PI/SiO_2_ and PI/NH_2_-POSS/SiO_2_ after sliding against GCr15 under different working environments, respectively. It was clearly seen that pure PI was more severely worn than other samples in air, vacuum environment, and vacuum atomic oxygen environment (Figure 7(a–a2)). Regarding PI/NH_2_-POSS, PI/SiO_2_ and PI/NH_2_-POSS/SiO_2_ (Figure 7a–d), the wear seemed more serious in air, which corresponded to the high wear rates. In vacuum and vacuum atomic oxygen environments (Figure 7(b–b2) and Figure 7(c–c2)), the wear scars of PI/NH_2_-POSS, PI/SiO_2_ and PI/NH_2_-POSS/SiO_2_ become slight and there were almost no deep furrows, because the abrasive particles were hardly accumulated on the sliding interfaces. In particular, the protective layer was generated from NH_2_-POSS and SiO_2_ in vacuum atomic oxygen environment, which endowed the surfaces of the samples with high hardness and further enhanced their wear resistance.

### 3.3. Analysis of Tribological Mechanisms

**Figure 8:**
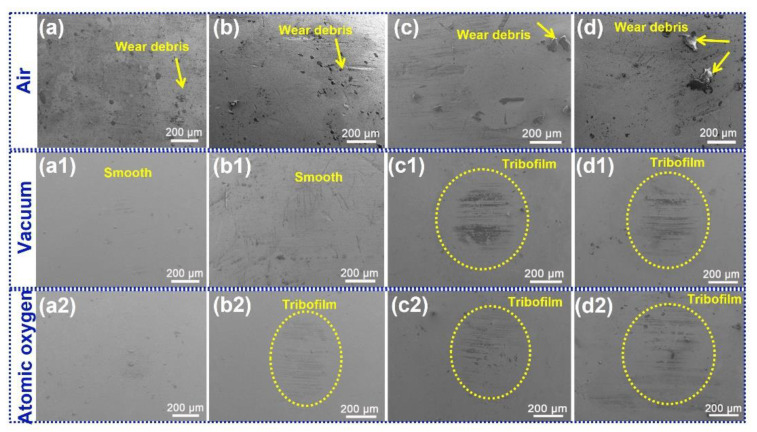
SEM images of the surfaces of the steel counterparts after they were rubbed with (**a**–**a2**) PI, (**b**–**b2**) PI/NH_2_-POSS, (**c**–**c2**) PI/SiO_2_ and (**d**–**d2**) PI/NH_2_-POSS/SiO_2_ in air, vacuum environment, and vacuum atomic oxygen environment, respectively.

Figure 8 displays images of the steel counterpart surfaces slid against PI, PI/NH_2_-POSS, PI/SiO_2_ and PI/NH_2_-POSS/SiO_2_ in air, vacuum environment, and vacuum atomic oxygen environment, respectively. Obviously, when sliding occurred in air, there were many wear debris accumulated on counterpart surfaces as indicated by the yellow arrows, which contributed to the unstable curves of the friction coefficients. Under vacuum and vacuum atomic oxygen environments, the counterpart surfaces were relatively clean and the wear debris was fed into the sliding zones and expedited the tribofilm growth. As shown in Figure 8(a1,a2), the tribofilms generated from PI were unnoticeable, which probably resulted in the direct contact of the sliding interfaces, and further aggravated the wear. The modification of polyimide can improve the bonding strength of the tribofilm with the counterpart. In particular, the patchy tribofilms were grown when PI/NH_2_-POSS, PI/SiO_2_ and PI/NH_2_-POSS/SiO_2_ were slid against GCr15 under vacuum and vacuum atomic oxygen environments. In a vacuum environment, the friction heat was hardly dissipated, adhesive friction occurred and the friction coefficient was increased. Nevertheless, the released nanoparticles could carry the load of the sliding interfaces and enhance the wear resistance. In atomic oxygen environment, the silicon-based protective layer was fed into the tribofilm and significantly increased the load-bearing capacity of the tribofilm. Thus, when the friction pair was exposed atomic oxygen environments, PI/NH_2_-POSS, PI/SiO_2_ and PI/NH_2_-POSS/SiO_2_ displayed the lower wear rate.

Table 2 shows the roughness of the counterpart balls after sliding process. Before tribological test, the Ra of the counterpart is approximately 326.82 nm. However, the Ra was significantly enhanced after the sliding process as shown in Table 2. In particular, the Ra of the counterpart balls after sliding against PI, PI/NH_2_-POSS, PI/SiO_2_ and PI/NH_2_-POSS/SiO_2_ in air was enhanced to 772.04, 741.85, 692.27 and 846.36 nm, respectively, which was higher than that obtained in vacuum and vacuum atomic oxygen. The results indicated that more wear debris are adhered to the counterpart surfaces when sliding occurred in air. In vacuum and vacuum atomic oxygen environment, the Ra of the counterpart displayed insignificant difference which corresponded to the morphologies of the counterparts in Figure 8. Moreover, it is found that the counterpart after sliding PI/NH_2_-POSS/SiO_2_ demonstrated the highest roughness; we assumed that the released SiO_2_ and NH_2_-POSS co-dominated the formation of the rough surfaces.

XPS analysis was used to determine the chemical states of the counterpart surfaces after sliding against PI, PI/NH_2_-POSS, PI/SiO_2_ and PI/NH_2_-POSS/SiO_2_ before and after AO attack. The intensity of the C element attached to the counterpart surfaces was almost unchanged before and after AO irradiation judging from the C 1s spectrum (Figure S2a,a1), indicating the little difference of polymeric materials contents in the tribofilms. From Fe 2p (Figure S2b), the Fe_2_O_3_ at 711.2 eV mainly dominated the chemical state of Fe when PI was slid against GCr15 in air and the peak at 725.1 eV corresponded to Fe_3_O_4_ [36]. Additionally, the Fe 2p peak at 712.6 eV was attributed to metal-organic compounds Fe(CO)x, indicating the chelation reaction between the transferred polymer with the steel counterpart occurred [31]. Nevertheless, when PI was sliding against GCr15 at vacuum atomic oxygen environment, the peak at 707.1 eV corresponding to Fe almost disappeared, indicating the counterpart was eroded by AO (Figure S2b1). Regarding the tribofilms generated from PI/NH_2_-POSS, PI/SiO_2_ and PI/NH_2_-POSS/SiO_2_, the chemical states of Fe element showed insignificant distinction in air and vacuum atomic oxygen environment. In Si 2p (Figure S2c,c1), Si-O in NH_2_-POSS appeared at 102.8 eV and Si-O in SiO_2_ confirmed at 103.4 eV, further illustrated the polymeric materials transferred into the counterpart. After AO attack, Si-O in the NH_2_-POSS was partially converted into Si-O in SiO_2_, judged from 103.4 eV (Figure S2c1). Additionally, Si(O)x at 103.2 eV was confirmed in the tribofilms of PI/SIO_2_ and PI/NH_2_-POSS/SiO_2_, indicating the formation of a protective layer after AO attack.

**Figure 9:**
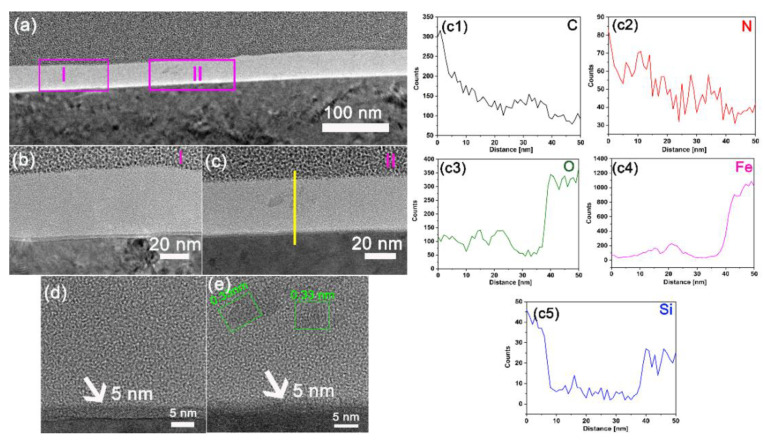
(**a**) Overview of TEM graph of FIB-cut cross-section of the tribofilm formed on the steel disk sliding against PI/NH_2_-POSS/SiO_2_ under atomic oxygen irradiation condition; (**b**) TEM graph of the zone I indicated in (**a**); (**c**) TEM graph of zone II indicated in (**a**); (**d**) HR-TEM graph of (**b**); (**e**) HR-TEM graph of (**c**); (**c1**–**c5**) EDS line of the yellow line indicated in (**c**).

In order to have a deep understanding of the nanostructure of the tribofilm formed on the counterpart surface, FIB-TEM analysis was conducted. As shown in Figure 9a, a tribofilm with a thickness of approximately 50 nm covers almost the entire surface of GCr15 when sliding against PI/NH_2_-POSS/SiO_2_ under atomic oxygen irradiation condition. Figure 9b,c shows the zone I and II indicated in Figure 9a. It is seen that the tribofilm consists of crystallization area and amorphous area, demonstrating the complex structure of the tribofilm.

**Figure 10:**
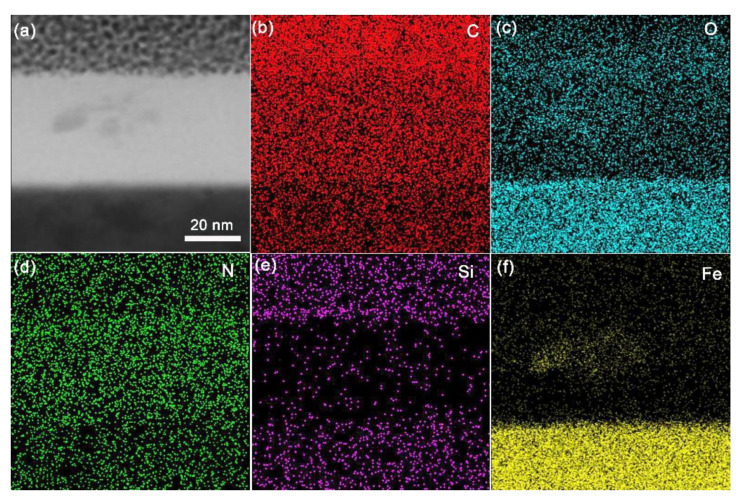
(**a**) TEM morphology of the tribofilm formed on the steel disk sliding against PI/NH_2_-POSS/SiO_2_ under atomic oxygen irradiation condition and the corresponding elemental maps of (**b**) C, (**c**) O, (**d**) N, (**e**) Si and (**f**) Fe.

Close inspection reveals that the tribofilm consists of two layers (Figure 9d,e). As indicated by the white arrows, the sublayer is adjacent to the steel substrate, the thickness of which is approximately 5 nm, composed mainly of Fe_3_O_4_ and Fe_2_O_3_ [37]. It is indicated that tribo-oxidation occurs at the initial sliding process. As seen from Figure 9e, above the sublayer, Fe_3_O_4_ (lattice spacing: 0.33 nm) was abraded from the oxidation layer and then compacted into the tribofilm. The EDS result along the yellow line in Figure 9c revealed that the amorphous matrix contained high fractions of C, N and O elements (Figure 9(c1–c3)), which originated from the transferred wear debris. Additionally, the elemental maps (C, O, N, Si and Fe) of the tribofilm around the yellow line in Figure 9c are displayed in Figure 10. It also can be found that the tribofilm is mainly composed of C, N and O. Moreover, Fe_3_O_4_ was further confirmed, as indicated by the high content O and Fe at the distance from 10 to 30 nm (Figure 9(c3,c4)), which could also be verified from the maps of O and Fe in Figure 10c,f. Meanwhile, high fractions of Si at the top layer of the tribofilm were verified by the EDS line in Figure 9(c5) and Figure 10e. It is suggested that an inert protective layer of Si was formed during the sliding process after AO attack, which inhibited the erosion to internal structure of the tribofilm.

**Figure 11:**
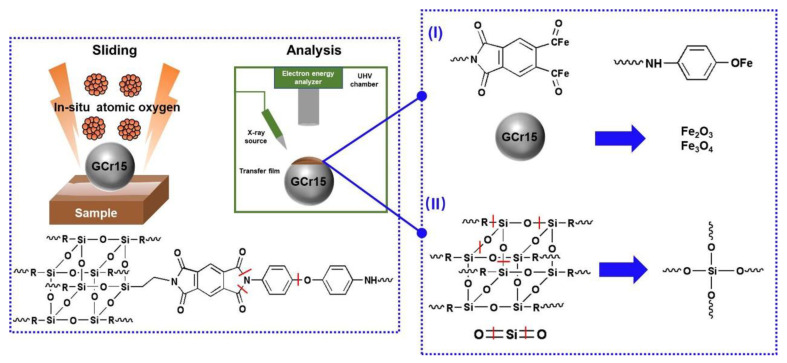
Schematic diagram of tribological mechanism of PI/NH_2_-POSS/SiO_2_ after sliding GCr15 under atomic oxygen irradiation condition.

Based on the analyses of FIB-TEM and XPS results about the structure and chemical state of the tribofilm generated from PI/NH_2_-POSS/SiO_2_, a tribofilm formation mechanism is proposed and summarized in Figure 11. The friction, shear, and atomic oxygen induced the breakage of C-O, C-N, and Si-O bond in the PI/NH_2_-POSS chains and led to the formation of a series of small molecules such as carbon-free radicals, oxygen-free radicals, Si(O)x and Si [38,39]. During the sliding process, the metal-organic compounds formed by the chelation of these organic molecular fragments and the iron in the counterpart (Figure 11I). Therefore, the bond between the tribofilm and the counterpart was enhanced, which endowed the tribofilm with a stiff structure. Moreover, the counterpart surface was oxidized to Fe_2_O_3_ and Fe_3_O_4_ under AO irradiation. As shown in (Figure 11II), Si(O)x and Si was formed due to the breakage of NH_2_-POSS and SiO_2_ and contributed to the formation of the Si-based protective layer. In summary, the results showed that the protective tribofilm formed on the steel surface led to excellent wear resistance of the PI/NH_2_-POSS/SiO_2_ composite under atomic oxygen irradiation conditions. 

## 4. Conclusions

In the present work, silica nanoparticles were added in situ into 3-amino-polyhedral oligomeric silsesquioxane modified polyimide molecular and their role in tribological performance of the composites were systematically investigated subjected to vacuum atomic oxygen environment. It is obviously evidenced that the addition of SiO_2_ particles and modified with NH_2_-POSS can improve the nano-mechanical properties when polyimide composites are exposed to atomic oxygen environment. Morphology characterization and XPS analysis indicated that the structure and the chemical state were changed, induced by AO attack. However, the friction coefficient in vacuum and vacuum atomic oxygen environments was higher than that in air, because the friction heat was difficult to diffuse in vacuum environment, leading to the adhesive friction. Nevertheless, the wear rates were significantly decreased under AO environment. It is believed that the tribofilm fed by the protective layer increased the load-carrying capability of the sliding interfaces, which enhanced the wear resistance of polyimide composites in atomic oxygen environment. In summary, it is expected that the outputs of this work can provide guidance for formulating PI-based tribomaterials used at space environment.

## Figures and Tables

**Table 1:** Designations and detailed compositions (wt%) of PI samples.

	PI	NH_2_-POSS	SiO_2_
PI	100	/	/
PI/NH_2_-POSS	99	1	/
PI/SiO_2_	99	/	1
PI/NH_2_-POSS/SiO_2_	98	1	1

**Table 2:** The roughness of the GCr15 balls after sliding against PI, PI/NH_2_-POSS, PI/SiO_2_ and PI/NH_2_-POSS/SiO_2_ under different working conditions (nm).

	Air	Vacuum	Vacuum Atomic Oxygen
PI	772.04	576.85	597.33
PI/NH_2_-POSS	741.85	645.82	615.79
PI/SiO_2_	692.27	673.53	633.04
PI/NH_2_-POSS/SiO_2_	846.36	711.45	720.36

## Data Availability

The data that support the findings of this study are available on request from the corresponding author.

## Supplementary Materials

The following supporting information can be downloaded at: https://www.mdpi.com/article/10.3390/polym15102385/s1. Figure S1. The schematic of vacuum tribometer equipped with in-situ AO irradiation; Figure S2. XPS spectra of (a) and (a1) C 1s, (b) and (b1) Fe 2p, and (c) and (c1) Si 2p of the counterpart surfaces after sliding against PI, PI/NH2-POSS, PI/SiO2 and PI/NH2-POSS/SiO2 in air and vacuum atomic oxygen environment, respectively.
