# Supplementary material for: High Wear Resistance of POSS Grafted-Polyimide/Silica Composites under Atomic Oxygen Conditions
Source: Polymers (Basel). 2023 May 19;15(10):2385. doi: 10.3390/polym15102385 (PMC10223808; doi:10.3390/polym15102385)
Supplement: Supplementary file 1 [file polymers-15-02385-s001.zip › polymers-2398208-supplementary.pdf]

Supporting Information

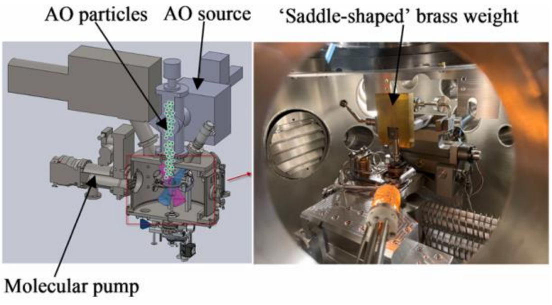

Figure S1. The schematic of vacuum tribometer equipped with in-situ AO irradiation.

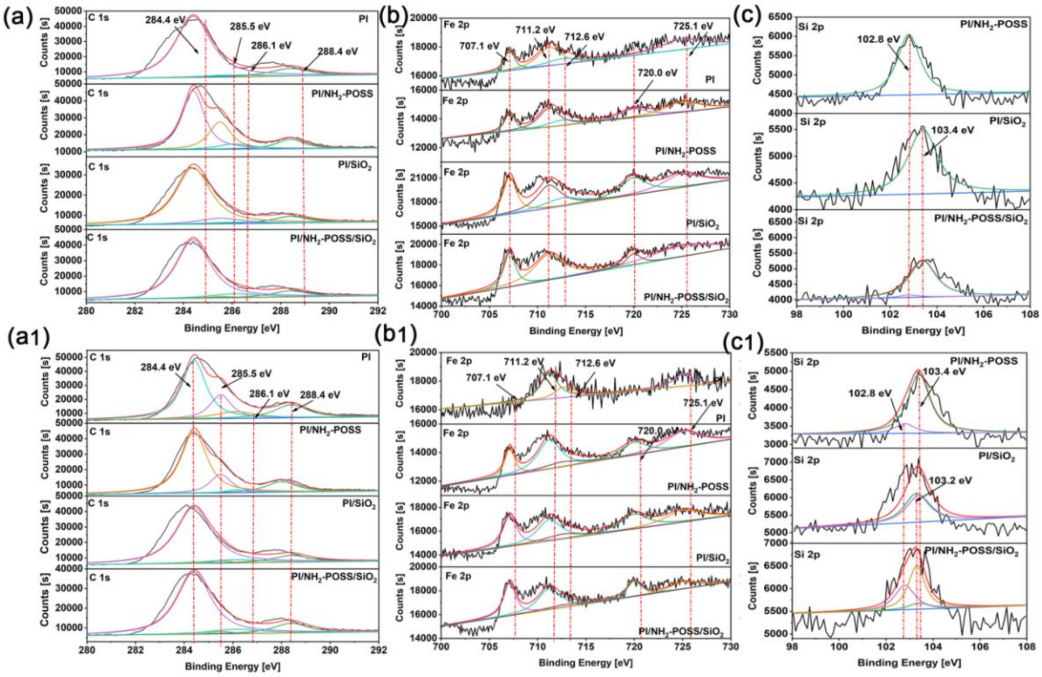

Figure S2. XPS spectra of (a) and (a1) C 1s, (b) and (b1) Fe 2p, and (c) and (c1) Si 2p of the counterpart surfaces after sliding against PI, PI/NH2-POSS, PI/SiO2 and PI/NH2-POSS/SiO2 in air and vacuum atomic oxygen environment, respectively.
